# Polymorphic Light Eruption: What's New in Pathogenesis and Management

**DOI:** 10.3389/fmed.2018.00252

**Published:** 2018-09-10

**Authors:** Serena Lembo, Annunziata Raimondo

**Affiliations:** ^1^Department of Medicine, Surgery and Dentistry, “Scuola Medica Salernitana”, University of Salerno, Salerno, Italy; ^2^Department of Clinical Medicine and Surgery, University of Naples, Federico II, Naples, Italy

**Keywords:** polymorphic light eruption, photosensitive disorders, phototherapy, apoptosis, delayed type hypersensitivity reaction

## Abstract

Polymorphic light eruption is the commonest photosensitive disorder, characterized by an intermittent eruption of non-scarring erythematous papules, vesicles or plaques that develop within hours of ultraviolet radiation exposure of patient skin. Together with the lesions, a terrible itch starts and increases with the spreading of the disease, sometimes aggravated by a sort of burning sensation. Clinical picture and symptoms can improve during the rest of the summer with further solar exposures. In the last years many advances have been performed in the knowledge of its pathogenesis and some news have been proposed as preventive, as well as therapeutic options. All this has been discussed in the current mini review.

## Introduction

During winter, sometimes dermatologists receive asymptomatic patients, with no specific lesions other than, perhaps, some post-inflammatory discoloration, but with a desperate need for help. They start to tell the story of a number of papules or vesicles appearing on their skin after the first intense sun exposure of the year. Together with the lesions, a terrible itch starts and increases with the spreading of the disease, following the next sun exposure, sometimes aggravated by a sort of burning sensation. Nevertheless, this people are model sun seekers and continue to enjoy the sunshine throughout the summer, waiting for the papules to gradually fade and disappear. The main questions are: how can I prevent this? Why I'm getting this problem since “5” years now, but I never had it before?

We are most probably dealing with polymorphic light eruption (PLE) and, following the requests of our patients, medical research has mainly been focused on prevention strategies become nowadays quite satisfactory. On the other side, the second and certainly less explored question remains unclear, unless multiple pieces have been added to this rather complicate puzzle. Aim of this brief review is to resume the most recent advances in PLE possible mechanisms and the most used protocols for prevention or treatment.

## Pathophisiology of polymorphic light eruption: what's new?

**Figure 1:**
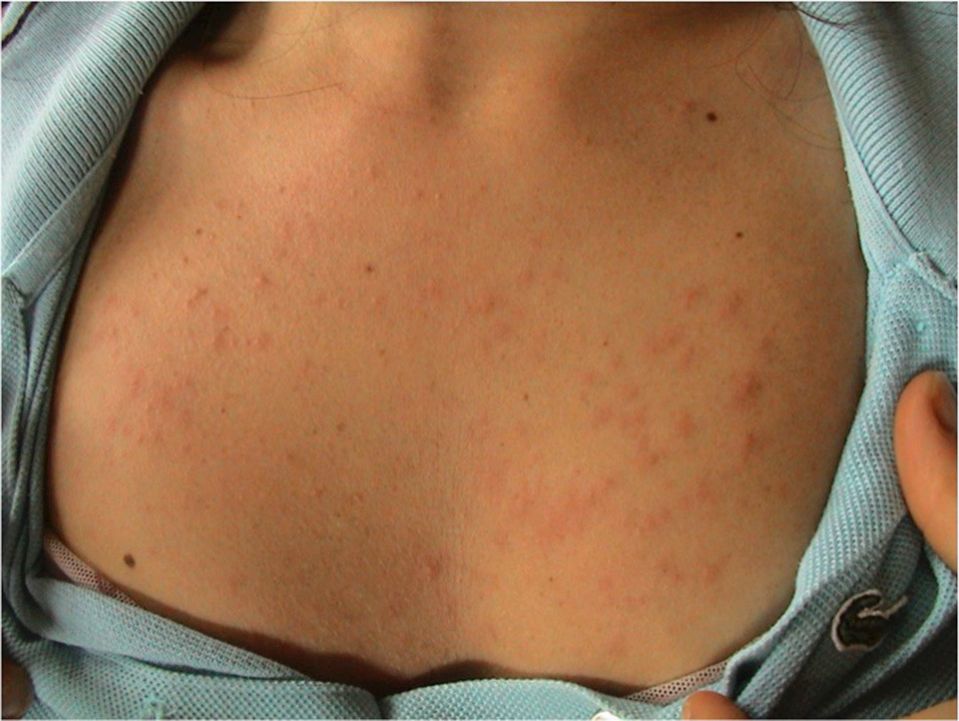
Clinical picture of polymorphic light eruption in a young woman.

PLE is the commonest photosensitive disorder, characterized by an intermittent eruption of non-scarring pruritic erythematous papules, vesicles or plaques (Figure 1) that develop within hours of ultraviolet radiation (UVR) exposure of patient skin. The disease is dependent on genetic susceptibility, as well as environmental component, such as type of exposure. PLE appears to cluster in families: it has been estimated that the prevalence of PLE was 21 and 18% in monozygotic and dizygotic twins, respectively (1). Moreover, a positive family history of PLE in first-degree relatives was present in 12% of affected twin pairs respect to 4% of unaffected twin pairs (*p* < 0.0001). The probandwise concordance in monozygotic was superior than in dizygotic twin pairs (0.72 vs. 0.30, respectively), demonstrating a strong genetic effect (1). Many genes of potential interest in the pathogenesis of PLE have been investigated with generally unrewarding results. Using segregation analysis, it has been estimated that 72% of the UK population carry a low penetrance PLE susceptibility allele (2).

### The failure of apoptosis: the possible photo-induced neo antigens

In a recent genome-wide expression analysis, only 16 genes were differentially expressed between PLE and healthy controls after UV irradiation respect to control (3). Of these genes, 14 showed lower expression in PLE patients, whereas two resulted over-expressed. Among the 14 genes with lower expression in PLE are: complement 1s subunit (C1s), scavenger receptor B1 (SCARB1) fibronectin (FN1), immunoglobulin superfamily member 3 (IGSF3), caspase-1 (CASP1) and paraoxonase 2 (PON2), all genes associated with apoptotic cell clearance. It has been supposed that protein modification during apoptotic cell clearance could lead to potential auto-antigen formation (4). Then, the reduced expression in PLE patients of genes connected to this process might represent a possible auto-antigen source, as well as a crucial phase in the initiation of the autoimmune process that promotes the disease (3). In accordance to these findings, Kuhn et al. showed accumulation of apoptotic cells in PLE patients irradiated either with 1.5 Minimal Erythema Dose (MED) of UVB, or 60–100 J/cm^2^ of UVA1, compared to controls (5).

### Immunity: tolerance's failure

Auto-antigens deriving from the inefficient clearance of apoptotic cells, are probably taken up by dendritic cells (DCs) and presented to naive T-cells (cytotoxic and helper) thereafter transformed in auto-reactive T cells (6, 7). This partial failure of the apoptosis contributes, together with the inadequate immunosuppression after UV exposure, to the antigen recognition and presentation, leading to the clinical manifestation typical of PLE patients (8). Indeed, the failure of normal UVR-induced immunosuppression has been proved as the main immunological abnormality in PLE, explained, initially, by the permanence of Langerhans cells (LCs) in the epidermis. This over-activation of the immune system, which escapes to the functional UV-induced tolerance, is probably responsible for the reduced skin cancer prevalence in PLE patients (9). On the other hand, the same mechanism is guilty for the failure of allergic contact dermatitis (ACD) suppression, after UVR exposure (10).

### Inflammatory pathway: delayed-type hypersensitivity reaction

The immunological mechanisms involved in PLE, with mediators from the innate and adaptive immune system, are very similar, either from the histological or the biochemical point of view, to the ACD ones. In effect, in the early seventies, Epstein first indicated PLE as a delayed-type hypersensitivity reaction (DTHR) to undefined UVR-induced cutaneous antigen (11). Recently, to reinforce this concept, some of the inflammatory mediators involved in ACD have been demonstrated also in PLE. For example, IL-1 family (12, 13), a growing group of cytokines that play several roles in immune regulation and inflammation (14), involved also in ACD pathogenesis (12, 15), has been explored also in PLE (16). IL-36α and IL-36γ, the pro-inflammatory members of IL-1 family were increased in PLE respect to controls, as for ACD samples, but IL-36γ was much enhanced in PLE than in ACD (16). Acting through the common receptor composed of IL-36R and IL-1R/AcP (IL-1RL2), IL-36α, IL-36β, and IL-36γ activate NF-κB and MAPKs, promoting inflammatory reactions. The increase of IL-36s in skin and peripheral blood of PLE patients indicates the activation of local and systemic immune response, as found in multiple inflammatory skin conditions (15, 17, 18). Probably, the link between IL-36s and UVR exposure is represented by the paracrine pro-inflammatory signal of toll like receptor (TLR)-3 activation, due to the release of RNA by necrotic keratinocytes (19). Indeed, the failure of apoptotic clearance in PLE, with abundance of cellular debris, could be responsible for an amplification of this “alert signal.”

Moreover, IL-36s could contribute to amplify the innate immune signal and the consequent inflammatory cascade, promoting antimicrobial peptides (AMPs) (20).

### Inflammatory pathway: AMPs and microbiome

As largely examined in multiple skin inflammatory processes, these mediators, named as defensins (α and β), cathelicidin (LL37), ribonuclease 7 (RNase7) and psoriasin (S100A7), in light of the imbalance induced by UVR on keratinocytes and skin microbiome, have also been investigated in PLE (21, 22). Patra et al. have found that the expression of psoriasin, RNase7, HBD-2, and LL-37 was increased in PLE lesional skin, whereas HBD-3 was decreased. Considering the skin surface as a “multiethnic world,” without forgetting the crucial role of keratinocytes, we can't exclude that AMPs release could be determined by modification in microbiome components after UV interaction (23). Indeed, microbiome could represent the source, direct or indirect, of the yet undetected UVR-induced antigens formed in PLE patients, leading to keratinocyte damage. As a consequence, LL-37, also induced by UVB, IFNγ, TNF-α, IL-6, could represent a potential indirect driver of PLE (23). It can form aggregates with self-nucleic acids able to activate pDCs: in psoriasis it has been recognized as the main autoantigen (24). Even though in PLE patients a complete absence of pDCs has been reported (25), an autoimmune milieu exists, and LL-37 could play a pivotal role, inducing other inflammatory pathways. In Figure S1 (Supplementary Material), the concepts expressed above are visualized in a cartoon.

### Therapy of polymorphic light eruption: what's new?

The first line of treatment for PLE includes sun avoidance, sunscreens and topical corticosteroids (26). For all patients preventive management is fundamental during sunny weather, by avoidance of intense UVR exposure and use of protective clothing, as well as application of sunscreen, in particular during the first exposure of the year. New generation broad-spectrum sunscreens, with high sun protection factor for UVB (SPF), together with longer wavelength UVA protection, have been reported to confer total or partial protection in up to 90% of PLE patients (27, 28). The use of oral antioxidants and nicotinamide could represent an additional valid preventive measure for these patients. The beneficial effects of nicotinamide have been investigated in an uncontrolled trial of 42 patients, where 60% of them reported complete abolition of symptoms when taking 2–3 g of nicotinamide daily, before sun exposure (29). Moreover, an extract of the tropical fern Polypodiumleucotomos [PL) has been shown to exert both potent antioxidant and immunomodulatory effects. When administrated at 480 mg/daily before sun exposure it significantly reduced skin reactions and subjective symptoms (30, 31). Regarding topical corticosteroid, even if no trials have been made to determine their efficacy in PLE, they are widely used to reduce itch (26). The second line of treatments for PLE includes systemic corticosteroids and photo(chemo)therapy (26). In a randomized, double-blind, placebo-controlled trial (32) the authors suggested the use of 25 mg prednisolone daily for 4–5 days at the onset of the eruption. Although, the potential long-term side effects of repeated courses of prednisolone must be considered, it could be advised for patients who suffer from occasional attacks of PLE, in the absence of any contraindications. In milder cases of PLE, a self-conditioning programme by graduate exposure to sunlight in springtime may be sufficient (33). Whereas, in more severe cases, medically supervised conditioning/desensitization treatment may be more appropriate. A course of psoralen and UVA therapy (PUVA), narrowband (NB)-UVB or broadband (BB)-UVB phototherapy, usually administered in early spring, can be effective as well as prophylactic treatment (26). Treatment protocol generally consists of one course of phototherapy/photochemotherapy over 5–6 weeks. Starting doses depend on minimal erythemal dose (MED) or minimum phototoxic dose (MPD), and are frequently 50–70% of these measured thresholds with incremental increases. To maintain the benefit acquired with the desensitizing therapy, a regular sun exposure throughout summer is advised, otherwise the hardening could be lost within 4–6 weeks. In the treatment of PLE, NB-UVB should be preferred to PUVA (strength of recommendation D; level of evidence 4), because of the lower risk of photocarcinogenesis, no risk of nausea or other side-effects associated with the ingestion of MOP, and no need to use post-treatment eye protection. However, PUVA should be considered, before other systemic treatments, if NB-UVB has failed or has previously triggered the eruption. In effect, as described below, the efficacy has been proved for multiple phototherapy regimens (BB-UVB, NB-UVB and PUVA), and side-effects, in term of rash provocation, erythema and itch were found to be more common with UVB than with PUVA (34). As summarized, in the literature, the efficacy of PUVA results in a 65–100% photoprotection rate (34). Multiple comparative studies have been performed, but from the only randomized controlled trial between PUVA and NB-UVB plus placebo tablets, three times a week, for 5 weeks, no significant difference in efficacy emerged, considering occurrence of PLE or outdoor activity restriction (35). In the 10 years retrospective review, reported by Man et al. (36), 170 patients with moderate-to-severe PLE received PUVA and/or UVB phototherapy. In detail, 8 patients received PUVA, 128 NB-UVB, 5 BB-UVB, and 29 patients, who failed to respond satisfactorily to NB-UVB, were given PUVA the following year. Self-assessments were made of the severity, and frequency of PLE episodes were reported at the follow up visits in autumn or during the following spring. Good or moderate improvement was reported in 88% of patients treated with PUVA and in 89% who received UVB. Of the patients treated with both PUVA and NB-UVB, the majority preferred PUVA. In another 14-years retrospective study on 79 patients treated with phototherapy (37), the efficacy, measured during the following summer in term of photoprotection with complete/partial remission, was 65% for PUVA, 82% for BB-UVB and 83% for UVA alone. In this case the treatment with PUVA was reserved to more severe PLE forms.

The mechanisms by which phototherapy induces photoprotection are not fully understood.

However, in the last years many advances have been performed. In addition to the well-known effects on melaninization and epidermal thickening of phototherapy, a wide range of UV induced immunomodulatory and anti-inflammatory properties are reported (38). Both UVB and UVA modulate adhesion molecule expression and induce soluble mediators, such as a-melanocyte-stimulating hormone, IL-10 (which suppresses the production of interferon γ) and prostaglandin E2, that explicate anti-inflammatory actions, preventing T cells activation and promoting apoptosis of skin infiltrating T cells (34). Moreover, it has been demonstrated that prophylactic UV photohardening in PLE patients restores the UV-induced LC migration from the epidermis to the skin-draining lymph nodes: one of the key cellular event in UV-immunosuppression (39). The tolerance induced by LC is mediated by the release of immunosuppressive cytokine such as IL-10, and by the interference with maturation and induction of regulatory T cells (Tregs) (40). Moreover, recently, an interesting link has been reported among LC, Tregs and vitamin D3. Indeed, it has been demonstrated that a short-term 1 week topical pre-treatment with the 1,25-dihydroxyvitamin D analogue, calcipotriol, diminished PLE symptoms after subsequent experimental photoprovocation (41). In addition, in a murine study 1,25-dihydroxyvitamin D showed comparable immunosuppressive effects as UV (42). Another interesting crosstalk has been highlighted between LCs and mast cells. In addition to their recognized role in atopy, dermal mast cells are also responsible for protecting the skin from UVB-induced inflammation, promoting UV immunosuppression (40). Human studies have demonstrated that after acute and chronic UVR exposure, dermal mast cells number increases, together with the release of IL-10. Overall these data suggest a potential role for mast cells in PLE, and in the mechanism of photohardening. In accordance with this, Wolf et al. have reported, for the first time, that photohardening significantly increases mast cell density in the papillary dermis of PLE patients (40). Summarizing, photohardening works in PLE by restoring the normal UV immune suppressive pathway, involving multiple cell types. The third line treatment for PLE includes the use of systemic immunosuppressive drugs, such as azathioprine and cyclosporine. However, only sporadic cases of patients successfully treated are reported in literature (43, 44). Moreover, hydroxychloroquine, omega-3 fatty acids, and beta-carotene have been proposed as treatments, but further double-blind, randomized controlled trials to really assess their clinical efficacy are required.

## Conclusions

Since the high prevalence and increasing incidence of PLE, associated to discomfort and life style restrictions, future studies are necessary to find novel therapeutic and/or preventive strategies. The choice of the appropriate PLE treatment requires a good knowledge of the individual clinical course of the disease together with the possibility of performing phototest. Some new aspects in the possible activation and promotion of the inflammatory process have been highlighted.

To the current state of knowledge, despite the identification of some crucial cellular regulation involved on the restoration of the immune tolerance, it is difficult to draw definite conclusions about the efficacy of various potential treatments in PLE, due to lack of adequate studies and the difficulty in assessing outcome measures. The clinical score to assess PLE severity (PLESI) (45) remains an instrument scarcely used and mainly restricted to research purposes. The deeper study of the underlying pathogenetic mechanisms of the disorder will permit a more targeted treatment approach.

## Author contributions

SL projected the manuscript, selected the material for the paper, wrote the initial draft and corrected the following drafts of the manuscript. AR was engaged in the writing of the manuscript, supporting new ideas of contents and style.

### Conflict of interest statement

The authors declare that the research was conducted in the absence of any commercial or financial relationships that could be construed as a potential conflict of interest.

## Supplementary material

Figure S1 Interplay between innate and adaptive immune system in a context of apoptosis failure in the epidermis. (Green symbol) Psoriasin: abundant expression in spinous and granular layers of PLE skin. (Blue symbol) RNase7: mainly expressed in keratinocytes of the stratum granulosum and stratum corneum of PLE lesions. (Yellow symbol) LL-37 was profoundly expressed in and around blood vessels and glands in PLE. (Violet cell symbol) Apoptotic keratinocytes with inefficient clearance.

## Abbreviations

IL: Interleukin
PLE: Polymorphic light eruption
ACD: Allergic contact dermatitis
DTHR: Delayed type hypersensitivity reaction
UVR: Ultraviolet radiation
C1s: Complement 1s subunit
SCARB1: Scavenger receptor B1
FN1: Fibronectin
IGSF3: Immunoglobulin superfamily member 3
CASP1: Caspase-1
PON2: Paraoxonase 2
MED: Minimal Erythema Dose
DCs: Dendritic cells
LCs: Langerhans cells
TLR: Toll like receptor
AMPs: Antimicrobial peptides
SPF: Sun protection factor
PUVA: Psoralen and UVA therapy
NB-UVB: Narrowband
BB-UVB: Broadband UVB
MPD: Minimum phototoxic dose
Tregs: Regulatory T cells
PL: Polypodiumleucotomos.
