# Supplementary figures and images for: Polymorphic Light Eruption: What's New in Pathogenesis and Management
Source: Front Med (Lausanne). 2018 Sep 10;5:252. doi: 10.3389/fmed.2018.00252 (PMC6139322; doi:10.3389/fmed.2018.00252)

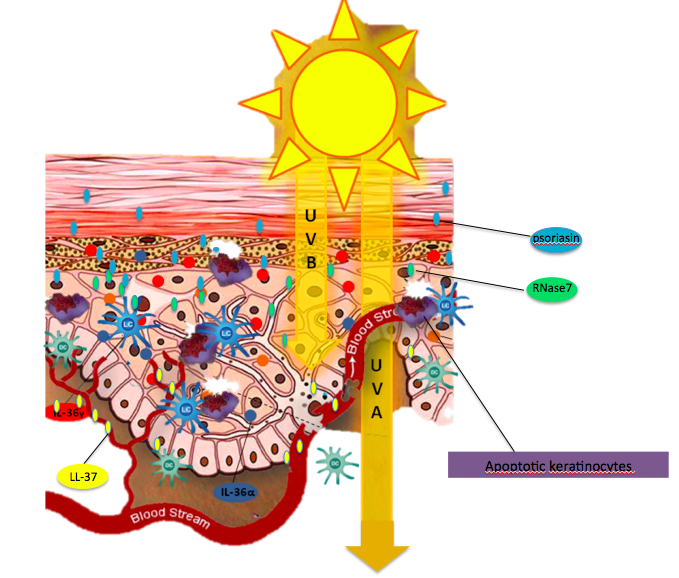

Supplement: Figure S1 — Interplay between innate and adaptive immune system in a context of apoptosis failure in the epidermis. (Green symbol) Psoriasin: abundant expression in spinous and granular layers of PLE skin. (Blue symbol) RNase7: mainly expressed in keratinocytes of the stratum granulosum and stratum corneum of PLE lesions. (Yellow symbol) LL-37 was profoundly expressed in and around blood vessels and glands in PLE. (Violet cell symbol) Apoptotic keratinocytes with inefficient clearance. [file Image_1.PNG]
